# Oesophageal Injury in Traumatic Pneumomediastinum: A Review of the Past 11 Years in an Upper Gastrointestinal Specialist Centre

**DOI:** 10.7759/cureus.30626

**Published:** 2022-10-24

**Authors:** Zak Shehata, Vinutha D Shetty

**Affiliations:** 1 Upper GI (Gastro-Intestinal) Surgery, Royal Preston Hospital, Preston, GBR

**Keywords:** acute care surgery and trauma, major trauma, upper gastrointestinal surgery, general trauma surgery, surgery of the oesophagus

## Abstract

Aims

Pneumomediastinum is a known radiological finding after major thoracic trauma; however, its significance and required investigative workup are not standardized. Furthermore, there is little evidence to suggest that traumatic pneumomediastinum is indicative of oesophageal injury. Our study sets out to investigate the incidence of oesophageal injury for these patients at our centre, and the relevant investigative workup is required.

Methods

Medical records were retrieved from our trust (Major Trauma Centre: 2012 to present, Upper Gastro-Intestinal (UGI) Centre: 2009 to present) to include trauma patients with radiological pneumomediastinum admitted between 2010 and 2021. Demographics, mechanism of injury, length of stay, and other significant findings were collected retrospectively using the electronic patient record.

Results

The data search retrieved 37 patients with traumatic pneumomediastinum. One patient was excluded due to incomplete records. Road traffic collisions were the most common presentation (18 patients), followed by falls (13 patients), penetrating trauma (three patients), assault (two patients), and workplace injury (one patient). The median length of stay was six days, with two inpatient deaths. One patient had a confirmed tracheobronchial injury on initial imaging which was managed conservatively, while six other patients underwent further oral contrast CT for suspected oesophageal injury. No patients in our dataset had a confirmed oesophageal injury.

Conclusion

Oesophageal injury is rarely seen in traumatic pneumomediastinum and is usually secondary to other chest injuries causing an air leak into the mediastinum. Oral contrast CT is the recommended investigation to exclude oesophageal injury.

## Introduction

Pneumomediastinum in the trauma setting is a finding often with unknown significance. Often patients complain of chest pain; however, up to 30% of patients present without any signs suggestive of pneumomediastinum [1]. Signs associated with this condition include subcutaneous emphysema, as well as the eponymous “Hamman’s sign” which describes crepitations in sync with the cardiac cycle [2].

The mediastinum runs throughout the length of the thorax and extends from the thoracic inlet to the superior border of the diaphragm between the two pleural cavities [3]. Typically, the mediastinum is split into four compartments: superior (trachea, oesophagus, aortic arch and great vessels, vagus nerve, superior vena cava, azygos vein, and thoracic duct); anterior (thymic remnants and internal thoracic arteries), middle (heart, trachea, and main bronchi), and posterior (oesophagus, descending aorta, thoracic duct, and sympathetic chain). Multiple communications between extrathoracic structures are present via tissue planes throughout the cranial and caudal aspects [4].

With the increased use of CT scanning in major trauma, incidental detection of pneumomediastinum has been on the rise [5]. Pneumomediastinum has been reported to occur in 10% of cases of chest trauma and is thought to be concerning for aerodigestive tract injury [6]. Despite the increased detection of pneumomediastinum, the incidence of tracheobronchial injury and oesophageal injury remains low, 1%-3% and <1%, respectively [7]. This differs depending on the mechanism of injury, with penetrating trauma having a more concerning likelihood of significant aerodigestive injury compared to blunt trauma [4]. Given the high mortality and morbidity associated with aerodigestive injury, an issue remains in determining how to correctly investigate these patients.

Lancashire Teaching Hospital Foundation Trust has been an upper gastrointestinal tertiary centre since 2009 and a major trauma centre since 2012. This report sets out to evaluate the experience of our centre in dealing with this complex radiological finding and to determine a safe and efficient method to rule out oesophageal injury in trauma patients with radiological pneumomediastinum. This article was previously presented as a poster at the 2022 ASIT Annual Conference on March 5, 2022.

## Materials and methods

Patient records were retrospectively identified by the business intelligence department from 2010 to 2021 at Lancashire Teaching Hospital Foundation Trust, the major trauma centre within Lancashire and South Cumbria’s Major Trauma Operational Delivery Network, receiving further referrals from four other trauma units. Patients were identified using the search term “pneumomediastinum”, International Classification of Diseases version 10 (ICD-10) code J98.2. Identified patients were cross-referenced with admission details and investigation results, and patients admitted with pneumomediastinum on either their CT or chest x-ray reported by a consultant radiologist were included. Patients admitted secondary to major trauma (either directly or indirectly via the major trauma network) were included. Patients who were deemed to have radiological findings of pneumomediastinum secondary to other causes such as COVID-19 or ventilation-associated barotrauma were excluded. Inpatient data were retrospectively analysed using the trust’s electronic patient record. Demographics, mechanism of injury, other significant radiological findings, further diagnostic workup, other associated injuries such as pneumothorax or flail chest, need for operative intervention, and patient outcomes including length of stay and mortality were recorded. Discharge letters were also reviewed to evaluate patient outcomes. Mortality was defined as death prior to hospital discharge.

Patient data were anonymised according to the trust’s information governance protocol to ensure patient confidentiality. For numerical variables, distribution was assessed using the Shapiro-Wilk test for normality. The mean was used to assess the average age; however, the median was used to assess the average length of stay, as these data were not normally distributed. Mann-Whitney U test was used to compare the median length of stay between the different mechanisms of injury and the effect of the presence of other radiological findings on the length of stay.

## Results

**Figure 1:**
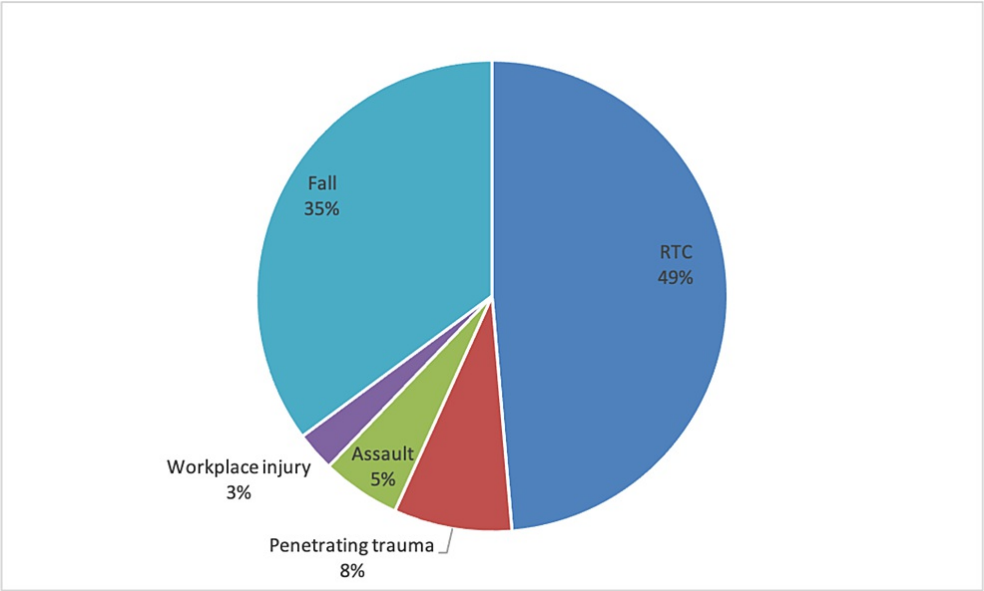
Distribution of mechanisms of injury within our dataset RTC: road traffic collision.

Our data search revealed 37 patients admitted to our trust between 2010 and 2021 with major trauma, which had pneumomediastinum on initial CT scanning. The average age of patients was 48 years old, with the majority being male (86.5%). In order of frequency, mechanisms of injury included road traffic collisions (RTCs) (18 patients), falls (13 patients), penetrating trauma (three patients), assault (two patients), and workplace injury (one patient). The distribution of mechanisms of injury can be seen in Figure 1. The most common investigation demonstrating pneumomediastinum was CT thorax/abdomen/pelvis (27 patients, 73%).

The most common simultaneous finding was pneumothorax (29 patients, 78.4%), followed by subcutaneous emphysema (24 patients, 64.9%). The frequencies of other common findings can be found in Table 1.

**Table 1:** Frequencies of simultaneous radiological findings

Radiological Finding	Number of Patients	Percentage of Total
Pneumothorax	29	76.3%
Subcutaneous emphysema	24	63.2%
Haemothorax	10	26.3%
Flail chest	9	23.7%
Sternal fracture	7	18.4%
Pneumoperitoneum	2	5.3%
Facial injuries	1	2.6%

None of the patients in the dataset had a confirmed oesophageal injury on initial cross-sectional imaging. Six patients underwent further investigation to exclude oesophageal injury. Five patients underwent a CT thorax with oral contrast, while two patients underwent a contrast swallow study. No patients within our dataset had a confirmed oesophageal injury after further investigation.

The median length of hospital stay for patients in our dataset was six days. The mortality rate for our dataset was 5.4% (two patients). One patient who had died suffered a suspected tracheobronchial injury (based on clinical grounds of consistent air leak from chest drain, oesophagogastroduodenoscopy (OGD) and bronchoscopy were negative), leading to severe cardio/respiratory instability and death. The second patient who died had presented with a traumatic cardiac arrest with 15 minutes of asystole. Their cause of death was hypoxic brain injury secondary to cardiac arrest.

The median length of stay was significantly greater in patients with CT findings of sternal fracture (U=54.5, p=0.049) and pneumothorax (U=62, p=0.047). Patients who suffered from pneumomediastinum from penetrating trauma had a significantly shorter length of stay (U=12.5, p=0.026). A full statistical comparison of the effects of CT findings on length of stay can be found in Tables 2, 3.

**Table 2:** Demonstration of statistical significance of radiological findings on length of stay

CT Findings	p Value
Sternal fracture	0.049
Flail chest	0.086
Subcutaneous emphysema	0.224
Pneumothorax	0.047
Haemothorax	0.257
Pneumoperitoneum	0.631

**Table 3:** Statistical analysis of the mechanism of injury and length of stay RTC: road traffic collision.

Mechanism of Injury	p Value
RTC	0.62
Fall	0.337
Penetrating trauma	0.026
Assault	0.769

## Discussion

There are multiple ways in which pneumomediastinum can develop in a trauma patient. The Macklin effect was first described in 1939, which describes a mechanism of increased intra-alveolar pressure leading to the rupture of alveoli [8]. The gaseous alveoli contents then track along bronchovascular sheaths and dissect into the thoracic hilum, leading to mediastinal emphysema [1,9]. As well as being secondary to blunt trauma, or trauma related to rapid deceleration, the Macklin effect can also occur in severe asthma exacerbations and positive pressure ventilation [1]. In the presence of pneumothorax, it has been suggested that pleural tears can lead to the tracking of air from the pleural space into the mediastinal cavity, causing pneumomediastinum [5]. Of course, pneumomediastinum in trauma patients can also occur in direct trauma to the aerodigestive tract leading to direct air leak, which is of great concern when this radiological diagnosis is made [1]. Traumatic oesophageal injury is usually caused by a penetrating mechanism such as a stabbing or gunshot wound [10]. Blunt injuries are associated with abdominal oesophageal injuries with blunt force with a full stomach, stretching the gastroesophageal junction leading to oesophageal tearing. Pneumomediastinum can also occur by gas tracking from other potential spaces. This can be seen as tracking from the abdomen secondary to hollow viscous perforation, as well as tracking from the cervical fascia in severe facial trauma [1]. The majority of our patients presented with the following blunt deceleration injuries: 18 road traffic collisions, 13 falls, and one workplace injury in which construction equipment failed and fell on the patient. While it is true that these incidents in themselves could technically cause a direct injury to the oesophagus, it is far more likely that for some of these patients, their pneumomediastinum was secondary to the Macklin effect. Furthermore, given that 78.7% of our patients also had a pneumothorax present, it may be the case that many of these findings could be due to tears in the pleura rather than injury to mediastinal structures. Within our dataset, two patients had pneumoperitoneum on their trauma CT scan, and one of these patients went on to have a contrast swallow study to rule out an oesophageal injury as the cause for their findings, which was negative. One patient had severe facial injuries, and it may be the case that air tracking from the fascial planes of the neck led to pneumomediastinum. This patient did not undergo any further diagnostic workup to exclude an oesophageal perforation.

Chouliaras et al. [6] investigated the incidence of aerodigestive injury in blunt thoracic trauma between 2007 and 2012 in California, USA. They identified 258 patients with pneumomediastinum, with 21 receiving further investigation to identify the potential aerodigestive injury. Of these, only one patient was shown to have an oesophageal injury on barium swallow, giving an incidence of 0.3%. This supports the fact that direct oesophageal injury after major trauma is a rare finding. On top of this, the oesophageal injury identified was successfully managed conservatively, which suggests that further diagnostic imaging did not necessarily lead to aggressive therapy. These findings are further compounded by the work of Lee et al. [11], who after analysis of 72 patients with pneumomediastinum from blunt trauma over nine years, found no instances of oesophageal injury. That being said, this study only specifically looked at patients following blunt trauma and did not include penetrating trauma within their dataset. A group in South Africa that evaluated 389 patients with incidental pneumomediastinum following blunt thoracic trauma found no incidence of aerodigestive injury within their patient group and suggest a conservative approach to patients with incidental pneumomediastinum in the absence of other complications [4]. The low incidence of oesophageal injury after blunt trauma may be secondary to the small size of the oesophagus, its anatomical location, and the extreme force required for direct injury leading to a high likelihood of fatal concurrent injuries [12].

**Figure 2:**
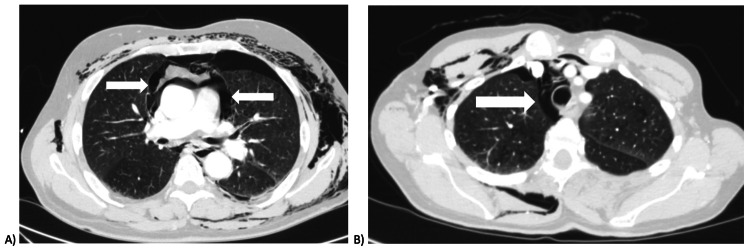
CT images of patients within our dataset Pneumomediastinum is highlighted by the white arrows. Note the subcutaneous emphysema and pneumothoraces in both patients (A, B).

There are multiple radiological signs that can hint at oesophageal injury in trauma patients, including mediastinal/subcutaneous air leak, oesophageal wall thickening, mediastinal abscesses, and leak of radio-opaque contrast [13]. CT with oral contrast has been shown to have a sensitivity of 59%-100% and specificity of 80%-100% in diagnosing oesophageal perforation [14]. Oral contrast CT has taken over from fluoroscopic oesophagography in the initial assessment of the oesophageal injury. In the trauma environment, it has the advantage of being available in the acute setting outside of normal working hours. Furthermore, CT has superiority over oesophagography in that it provides information on the trauma burden of other surrounding structures within the thoracic cavity. Given that in the case of oesophageal injury, delays in treatment have been significantly linked to greater mortality, it is of great value that oral contrast CT scanning can be conducted on an urgent basis [15]. The World Society of Emergency Surgery (WSES) guidelines [15] on oesophageal trauma suggest contrast-enhanced CT or CT oesophagography in stable patients who do not require immediate operative intervention. Figures 2A, 2B demonstrate the CT images of patients within our dataset. Both of these were the initial trauma CT without oral contrast. Of the six patients who underwent further diagnostic workup in our dataset, five of our patients underwent an oral contrast CT to successfully rule out oesophageal injury. One patient underwent both oral contrast CT and barium swallow, whereas another patient had an oesophageal injury ruled out on barium swallow alone. Given the potential urgency of diagnosis, we suggest urgent CT with oral contrast in patients with suspected traumatic oesophageal injury, in order to guide further urgent management.

Our dataset shows similar characteristics to other publications within the literature. As mentioned earlier, 78.4% of our patients also had a pneumothorax, which has also been reported as a common finding in traumatic pneumomediastinum patients [4,5]. Our median length of stay of six days was shorter than similar reports in the literature [5-7,11]. This may be due to the fact that our hospital is a tertiary upper gastrointestinal centre, and clinician expertise may lead to earlier discharge. Sternal fracture and pneumothorax were associated with a greater length of stay, which may reflect the greater total body trauma burden usually associated with sternal fractures and the time taken for pneumothoraces to be effectively treated in the context of a major trauma patient. Penetrating injuries have a significantly shorter length of stay, which may be due to the isolated injury associated with penetrating injury, rather than blunt polytrauma as seen more often in RTCs and falls. Mortality of patients with traumatic pneumomediastinum has been reported to range from 3% to 23% [4-7,11]. While our mortality rate of 5.4% is on the lower end of this spectrum, given the relatively small sample sizes of these studies, it is hard to delineate a significant difference.

Our study has a number of limitations. Firstly, due to the relatively low incidence of traumatic pneumomediastinum, our sample size is quite small, limiting the power of our observations. Furthermore, it is not known how many patients may have had an occult oesophageal injury which did not show on their initial trauma CT, which healed spontaneously. On top of this, our report is a single-centre study with retrospective analysis.

## Conclusions

Here, we show the experience of a major trauma and upper gastrointestinal tertiary centre in dealing with traumatic pneumomediastinum caused by both blunt and penetrating injuries over 11 years. This report confirms the low incidence of oesophageal injury in traumatic pneumomediastinum. This radiological finding is likely secondary to injuries to surrounding structures or the Macklin effect. Given its high sensitivity/specificity and its availability in the acute setting, we recommend the use of oral contrast CT scanning in the small subset of patients in which traumatic oesophageal injury is suspected.
